# Investigation of the Effects of Silicon Dioxide Nanoparticles and Environmental Contaminants on Immunocytotoxic and Antioxidant Defense Systems in Model Organism *Galleria mellonella* L.

**DOI:** 10.1007/s12011-025-04723-w

**Published:** 2025-06-26

**Authors:** Benay Tuncsoy, Murat Idikut, Mustafa Tuncsoy

**Affiliations:** 1 Department of Bioengineering, Engineering Faculty, Alparslan Turkes Science and Technology University, Adana, Turkey; 2 Department of Biology, Science and Letter Faculty, Cukurova University, Adana, Turkey

**Keywords:** *G. mellonella*, Silicon dioxide nanoparticle (SiO_2_ NPs), Cadmium sulfate (CdSO_4_), Abamectin

## Abstract

Silicon dioxide (SiO_2_) nanoparticles are chemically stable, biocompatible, abundant, and inexpensive. Moreover, they are highly surface-reactive. While these properties make them suitable for environmental applications, they also raise questions about their reactivity with environmental contaminants. This study investigated the enzyme activities of superoxide dismutase (SOD), catalase (CAT), glutathione peroxidase (GPx), cytochrome P450 (Cyt P450), glutathione S-transferase (GST), and acetylcholinesterase (AChE), as well as total differential hemocyte counts and apoptotic index in the hemolymph, midgut, and fat body of *Galleria mellonella*, were investigated following exposure to an LD_50_ value of SiO_2_ NPs (396 μg/mL), an environmental concentration of abamectin, cadmium sulfate (CdSO_4_), and copper sulfate (CuSO_4_), both singly and in a mixture. A decrease in the total hemocyte count was observed in the groups that were singly treated with SiO_2_ and CdSO_4_. However, an increase was observed in the SiO_2_ NPs + CdSO_4_ mixture, and a decrease was observed in the SiO_2_ NPs + abamectin group compared with the control. Treatment with SiO_2_ NPs, CdSO_4_, and abamectin singly and in mixture altered the levels of prohemocytes, plasmatocytes, spherulocytes, granulocytes, and oenocytoids. This study determined that SiO_2_ NPs, CdSO_4_, and abamectin lead to toxic effects in *G. mellonella* larvae following single and mixture applications. It was also observed that SiO_2_ NPs may increase the toxic effects of environmental pollutants on the antioxidant defense and immune systems, depending on tissue differences following mixture applications.

## Introduction

Nowadays, nanotechnology has led to significant advances in many fields such as materials, electronic devices, medical applications, and energy production. The physicochemical features of NPs and nanomaterials, such as shape, physicochemical stability, chemical composition, size, surface energy, surface area, crystal structure, and surface, all have an effect on their toxicity indicators [1]. Furthermore, although people are becoming more aware of the potential effects of nanoparticles on human health and the environment, their commercial use is increasing, and concerns about their potential harmful effects are growing**.** Recently, several researches have focused on the toxicity impacts of diverse nanoparticles in order to discover different elements of nanotoxicity [2]. Humans are continually exposed to a variety of pollutants, including NPs, which are becoming more prevalent as a result of the rapid advancement of nanotechnologies and their unintentional or intentional discharge into the environment. Although these nanotechnological advances have enabled progress, their long-term effects on the environment and non-target organisms remain unclear. Moreover, their excessive use can lead to uncontrolled and excessive transfers to upper trophic levels [3]. Due to their special properties at the nanoscale, nanoparticles can interact physicochemically with organic chemicals or metals in the environment, changing their bioavailability and also causing other different reactions, including synergistic, antagonistic, and potentiating effects [4]. The ability of nanoparticles to adsorb an environmental pollutant is critical to the toxicity of the NP or pollutant, as it facilitates the entry of the pollutant into the body, particularly through the absorption of nanoparticle-pollutant complexes. Complexed pollutants can be released into the body after entering the cell, raising their concentration and, as a result, bioavailability and toxicity. This process is known as the Trojan Horse effect [4, 5]. This idea encourages the entrance of hazardous substances adsorbed on NPs, causing an increase in intracellular pollutant concentration. In other cases, the complexes are effectively swallowed, and toxicity can be reduced if pollutants desorb from nanoparticle-pollutant complexes in limited or partial amounts [5].

Silica nanoparticles (SiO_2_ NPs) are considered an appropriate material for biomedical applications and are utilized in biosensors, biomarkers, cancer therapy, and DNA/drug delivery [6–10] and are widely researched for enzyme immobilization [11]. Although SiO_2_ NPs are often regarded as biocompatible materials for biomedical and biotechnological applications, they can aggregate and remain in the kidney, heart, spleen, liver, and brain following ingestion, inhalation, or skin application [12]. It is thought that they may also have insecticidal effects by blocking the digestive system of the insect and causing malformation in its external morphology [13].

The presence of organic and inorganic pollutants in the environment causes deterioration, a severe issue that endangers the global ecosystem [14, 15]. Environmental pollutants can be defined as substances that harm the environment as a result of human activities and natural consequences. These pollutants include heavy metals and pesticides. Cadmium has become a pollutant in the environment because of industrial expansion and modern advances in technology [16]. The other material utilized in this study is abamectin. It is a natural substance derived from the soil bacterium *Streptomyces avermitilis* that is a widely used insecticide and anthelmintic. Abamectin affects the nervous system of insects, causing fatal consequences. It specifically targets glutamate-gated chloride channels and gamma-aminobutyric acid (GABA) gated chloride channels [17].

*Galleria mellonella* larvae have an immune system that is similar to mammals’ innate immune response, and in recent years, they have been employed as model organisms to study the virulence mechanisms of human pathogens [18–20]. *G. mellonella* larvae have a wide surface area and are easy to isolate hemolymph, making them ideal for immunological research [21]. They may be cultivated in great quantities in the laboratory without the need for specialized equipment [22]. Furthermore, *G. mellonella* can live at temperatures ranging from 25 to 37 °C, making it useful for infection investigations and allowing experiments to simulate mammalian systems [23, 24].

Pollutant-nanoparticle interactions, as well as synergistic toxicity, are often overlooked. The goal of this research is to give a review of the combined toxicity of NPs and co-pollutants in order to emphasize the lack of evidence in the current literature and suggest that this issue deserves immediate attention. However, NPs released indiscriminately into the environment may interact with or absorb other pollutants on their surfaces, allowing them to enter the body [18]. This may cause a significant alteration in the toxicological profile. As a result, while these interactions are difficult to characterize, they need serious consideration. This study demonstrates that the impact of the absorption effects of SiO_2_ NPs on environmental contaminants by analyzing the antioxidant and immune system of model organism *G. mellonella*.

## Materials and Methods

### Materials

The following materials were purchased from Sigma Aldrich (St. Louis, MO, USA): SiO_2_ NPs (nanopowder (spherical, porous), 5–20 nm particle size (TEM), 99.5% trace metals basis), CdSO_4_ (≥ 99.99% trace metals basis), 4-nitroanisole (PNOD), 3,4-dihydroxy L-phenylalanine (L-DOPA), Brilliant Blue R Concentrate, entellan, ethidium bromide, Triton and CDNB. Giemsa’s Azure Eosine and Methylene Blue Solution was purchased from MERCK, and abamectin was purchased from Syngenta (Agrimec®EC, Syngenta®; analytical standard). Sephadex® G-25 (PD10, Pharmacia).

### Determining the LD50 value

*G. mellonella* larvae were reared at 30 ± 1 °C, 65 ± 5% RH on a diet composed of bran, honey, glycerol, honeycomb, and distilled water [24]. Newly hatched larvae were reared through the last instar on the artificial diet. The last instar *G. mellonella* larvae were removed from the diet medium and separated into control and treatment groups. The larvae were injected with various concentrations of 50, 100, 250, 400, and 500 μg/mL SiO_2_ NPs using a Hamilton injector to determine the LD_50_ value of SiO_2_ NPs. Concentrations were determined according to previous studies. After treatment, the last instar larvae were placed in petri dishes (*n* = 10). The total number of dead larvae in the treated groups was recorded 120 h after the application of the SiO_2_ NPs. Then, the LD_50_ values of SiO_2_ NPs for the last instar of *G. mellonella* were determined using the probit analysis method by SPSS 21 statistical data software.

### Experimental Design and Antioxidant Enzyme Activities

**Fig. 1:**
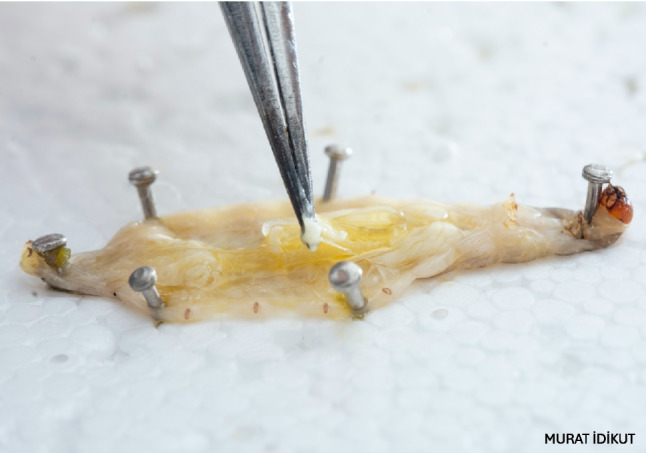
Dissection of *G. mellonella*

For the experiment, the LD_50_ value of SiO_2_ NPs (396 μg/mL), the environmental concentration of CdSO_4_ (10 µg/L), CuSO_4_ (10 µg/L), and abamectin (Agrimec®EC, Syngenta®; analytical standard) (10 µg/L) was used singly and in mixtures. The experimental period was determined as 120 h according to the LD_50_ analysis. After the injection of SiO_2_ NPs, CdSO_4_, CuSO_4_, and abamectin singly and in mixtures, the larvae were placed in petri dishes for 120 h. Then, they were put on ice for 2–3 min to slow their movements and cleaned with 95% ethyl alcohol. The larvae were dissected with micro scissors, and the fat body and midgut were placed in Eppendorf tubes with cold homogenization buffer (20 mM; pH 7.6) (Fig. 1) and homogenized at 4 °C by Ultra Torrax. The fat body was homogenized with an ultrasonic homogenizer (Bandelin Sonoplus. HD 2070, Berlin, Germany) at 50 W, 40–50 s in homogenization buffer. The homogenates were centrifuged at 500 × g for 15 min (+ 4 °C), and supernatants were recentrifuged at 12,000 × g for 45 min (4 °C) to participate in the mitochondrial fraction. The cytosolic fraction was purified on a Sephadex® G-25 (PD10, Pharmacia) gel column to remove low molecular weight proteins [25]. The samples for biochemical assays were frozen at − 80 °C until use. The methods to be used for the determination of the antioxidant enzyme activities are described by Sezer Tuncsoy et al. [26].

### GST Activity

GST activity was determined using the method developed by Habig et al. [27] based on conjugating CDNB with reduced glutathione [28].

### AChE Activity

As for AChE activity, the midgut and fat body were homogenized on ice in five volumes of a Tris–HCl buffer (100 mM, pH 8.0) containing 10% Triton and centrifuged at 12,000 × g for 30 min (4 °C). The AChE activity was assayed as described by Ellman et al. [29].

### Cyt P450 Activity

4-Nitroanisole (PNOD) was utilized as a substrate to determine cytochrome P450 monooxygenase enzyme activity [30].

### Phenoloxidase Activity

Eight microliters of hemolymph and 400 µL of ice-cold phosphate buffered saline (PBS, pH 7.4) are mixed in an Eppendorf tube. Then, it was centrifuged at 10,000 g for 5 min at 4 °C. The supernatant was mixed with 3,4-dihydroxy L-phenylalanine (L-DOPA), and the mixture was incubated for 20 min at 25 °C. Then, read the absorbance at 490 nm at 5-min intervals between 0 and 30 min in a UV spectrophotometer. The data obtained were determined as U/mg protein/min [31].

### Protein Content

Protein content was measured according to the Bradford [32] method using bovine serum albumin as a substrate.

### Total and Differential Hemocyte Counts (THC and DHC)

After 120 h, *G. mellonella* larvae were placed at − 20 °C for 15–20 s to stop their movements. The larvae were sterilized with 95% ethanol and cut from the first proleg to obtain hemolymph. Four microliters of hemolymph was transferred to an Eppendorf tube containing 36 μL of anticoagulant (0.186 M NaCl, 0.017 M Na_2_EDTA, 0.098 M NaOH and 0.041 M citric acid, pH 4.5). 1:10 μL of the 10% diluted cell solution was added to the Neubauer hemocytometer. The hemocytes were counted using a Leica DM750 microscope and the count of hemocytes per mL of hemolymph was determined using the Jones method [33]. As for DHC, 5 µL hemolymph was collected using a micropipette, then spread on slides and led to dry at room temperature. Air dried smear was then fixed in neat alcohol for 10 min, stained with Giemsa stain (MERCK Giemsa’s Azure Eosin and Methylene Blue Solution) and mounted in Entellan (Sigma Aldrich, Darmstadt, Germany). The hemocyte types were identified and counted using a Leica DM750 microscope.

### Apoptotic Index

The larvae were held at − 20 °C for 15–20 s to inhibit movement for dissection of hemolymph. After sterilization with 95% ethanol, 5 µL of the hemolymph was collected into the Eppendorf tubes contained 5 µL AO and 5 µL EB and mixed thoroughly. Then, 5 µL of the mixture was applied to slides cleaned with 70% ethyl alcohol. Slides were allowed to dry for 1–2 min before examination under a fluorescence microscope under a blue filter [34].

### Data Analysis

Statistical data were compared between the control and experimental groups using the Student Newman Keul’s (SNK) test in the SPSS 21 programme. A *p*-value of < 0.05 is considered statistically significant.

## Results

### Antioxidant Enzyme Activities

**Fig. 2:**
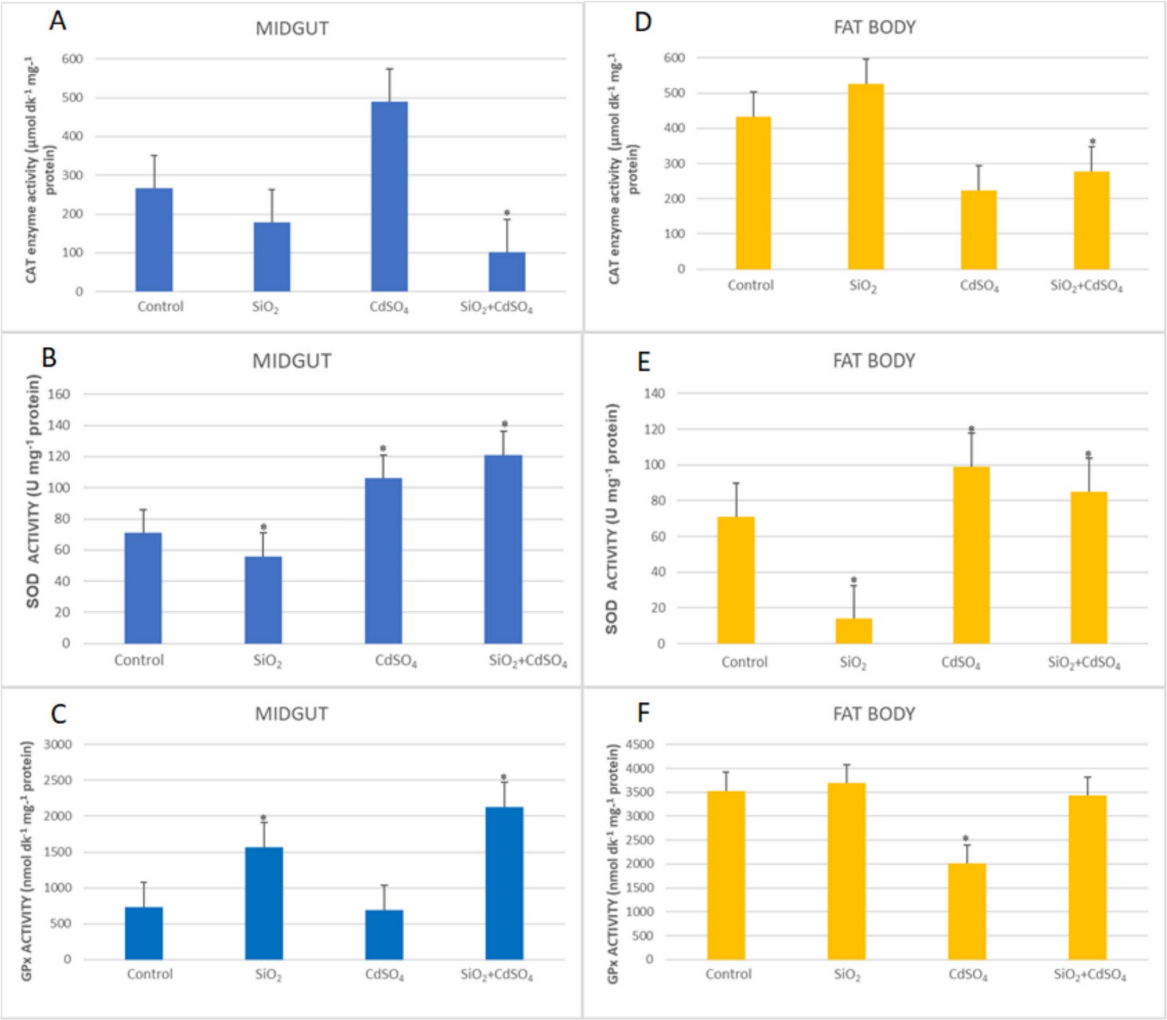
CAT, SOD, and GPx enzyme activities in the midgut (**A–C**) and fat body (**D–F**) of *G. mellonella* larvae were exposed to SiO_2_ NPs and CdSO_4_ singly and in mixtures (*SNK indicates that there is a statistically notable variation (*p* < 0.05))

The effects of SiO_2_ NPs and CdSO_4_ singly and in mixtures on the CAT, SOD, and GPx enzyme activities in the fat body and midgut of *G. mellonella* are presented in Fig. 2. There was an increase in CAT activity in the midgut in the mixture applied group according to the control, and this increase was found to be statistically significant (37.85-fold) (*p* < 0.05).

As for fat body, exposure to CdSO_4_ and mixture resulted in 1.94-fold and 1.55-fold decreases, respectively, while an increase occurred in the SiO_2_ NPs applied group (1.22-fold) (*p* < 0.05). There was a significant decrease in SOD activity in the midgut in the SiO_2_ NPs applied group compared to the control (1.25-fold; *p* < 0.05), while increases were observed in the CdSO_4_ and mixture applied groups. These increases resulted in 1.5-fold and 1.7-fold, respectively (*p* < 0.05). As for fat body, a decrease in SOD activity was observed in the SiO_2_ NPs applied group compared to the control, but increases were detected in the CdSO_4_ and mixture applied groups (1.44-fold and 1.24-fold, respectively) (*p* < 0.05). As for GPx activity, there was a decrease in GPx activity in the midgut in the SiO_2_ NPs applied groups compared to the control (2.13-fold), but an increase was observed in the mixture applied group (2.9-fold) (*p* < 0.05). As for fat body, a significant decrease in GPx activity was only detected in the CdSO_4_ applied group compared to the control (1.76-fold) (*p* < 0.05) (Fig. 2).

**Fig. 3:**
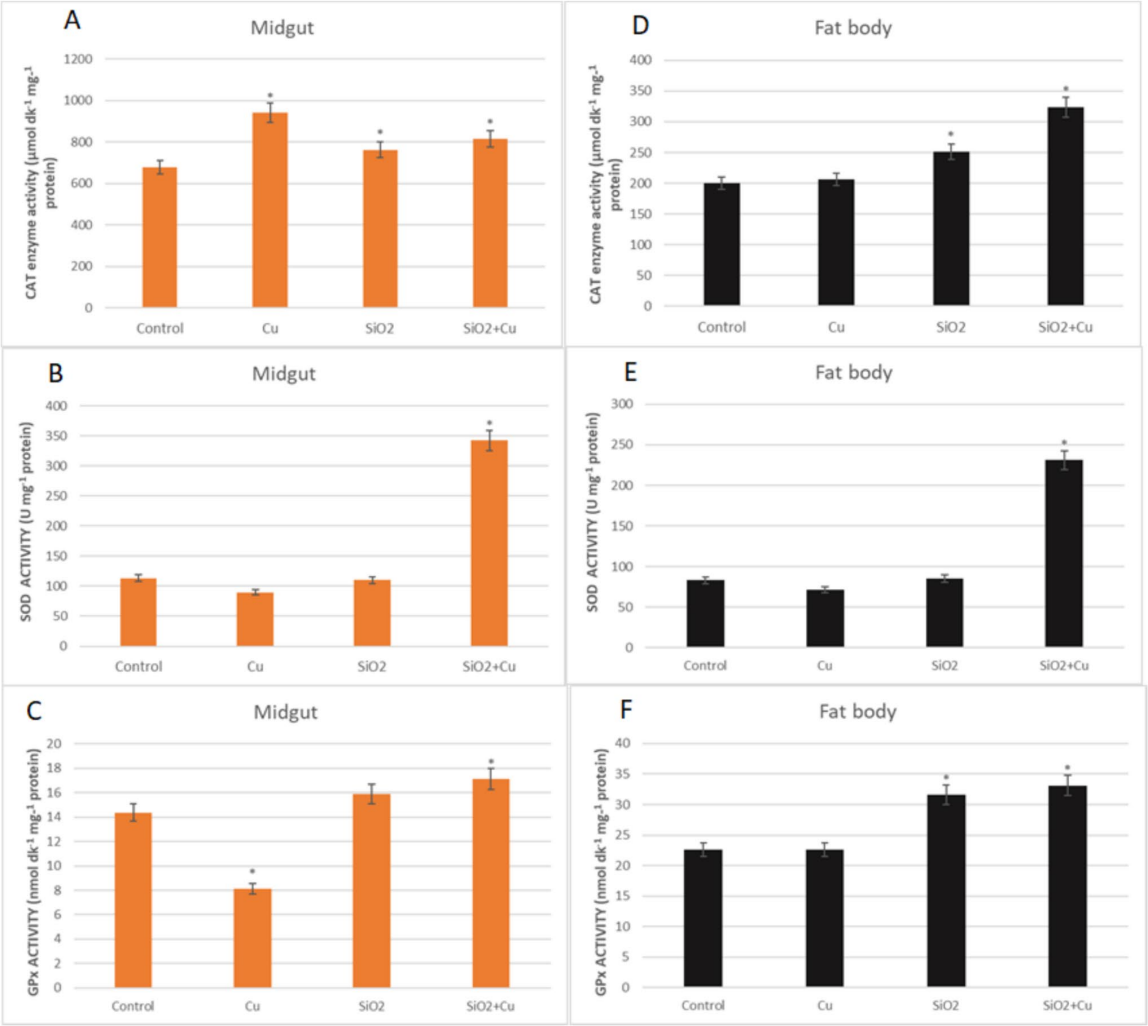
CAT, SOD, and GPx enzyme activities in the midgut (**A–C**) and fat body (**D–F**) of *G. mellonella* larvae were exposed to SiO_2_ NPs and CuSO_4_ singly and in mixtures with SiO_2_ NPs (*SNK indicates that there is a statistically notable variation (*p* < 0.05))

The effects of SiO_2_ NPs and CuSO_4_ singly and in mixtures on CAT, SOD, and GPx enzyme activities in the fat body and midgut of *G. mellonella* are presented in Fig. 3. While no statistical difference was observed in SOD activity in the midgut and fat body in the SiO_2_ NPs and CuSO_4_ applied groups compared to the control (*p* > 0.05), a significant increase was observed in the mixture applied groups (3.03-fold and 2.78-fold, respectively; *p* < 0.05). As for CAT activity, an increase was observed in the midgut of all treatment groups compared to the control (1.39-fold, 1.12-fold, and 1.20-fold, respectively; *p* < 0.05). In fat body, 1.26- and 1.62-fold increase in CAT enzyme activity was observed in SiO_2_ NPs and mixture applied groups, respectively (*p* < 0.05). In GPx activity, while there was a decrease in enzyme activity in the CuSO_4_ singly applied group in the midgut, a 1.19-fold increase was detected in the mixture applied group compared to the control (*p* < 0.05). In fat body, 1.47 and 1.40-fold increases in GPx enzyme activity were observed in SiO_2_ NPs singly and mixture applied groups, respectively (*p* < 0.05).

**Fig. 4:**
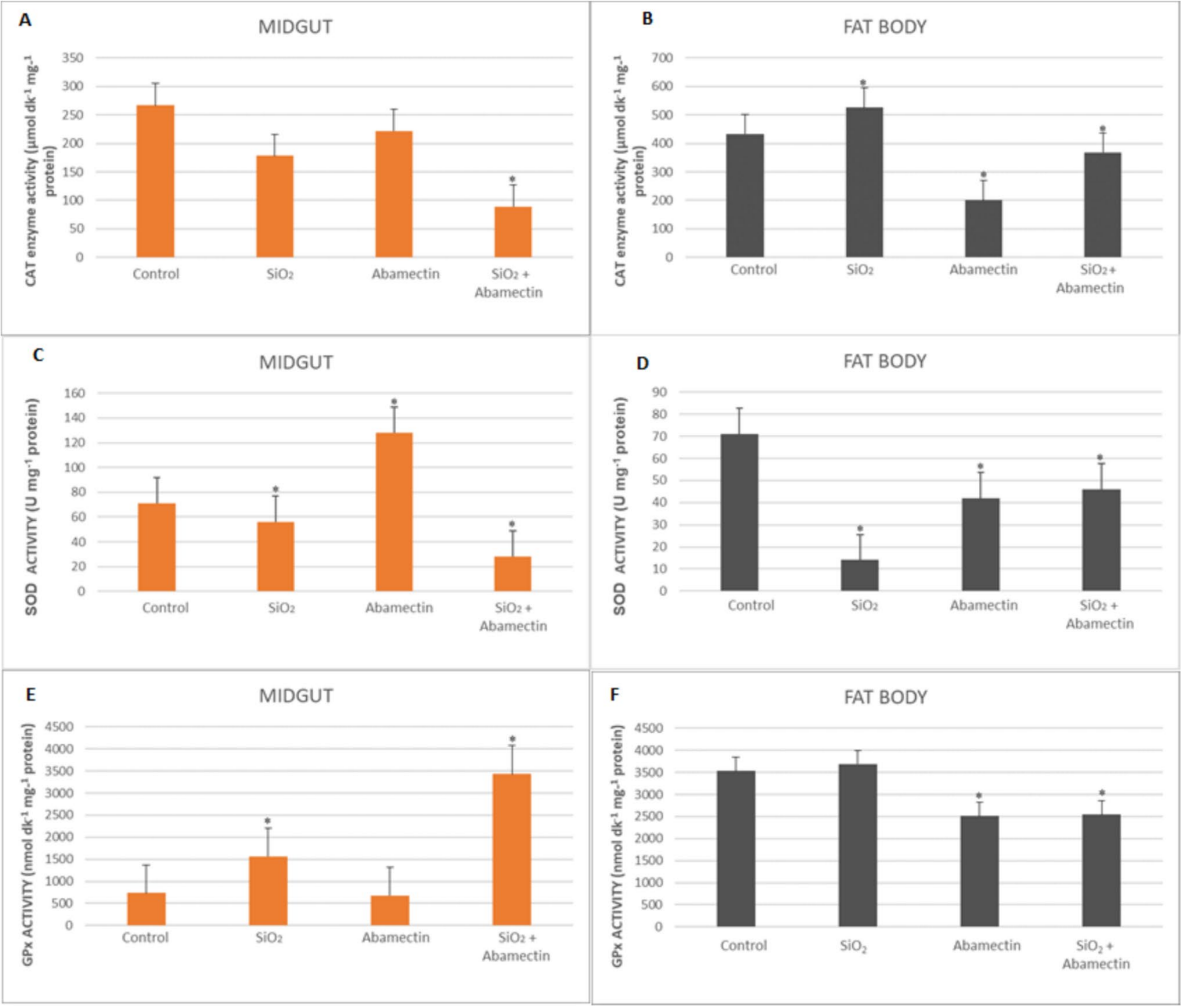
CAT, SOD, and GPx enzyme activities in the midgut and fat body of *G. mellonella* larvae were exposed to SiO_2_ NPs and abamectin singly and in mixtures with SiO_2_ NPs (*SNK indicates that there is a statistically notable variation (*p* < 0.05)

The effects SiO_2_ NPs and abamectin singly and in mixtures on CAT, SOD, and GPx enzyme activities in the fat body and midgut of *G. mellonella* are presented in Fig. 4. There was an increase in CAT activity in the midgut in the mixture applied group according to the control and this increase was found to be statistically significant (33.53-fold) (*p* < 0.05). The decreases in CAT activity in fat body was observed in the Abamectin and mixture applied groups, this decrease resulted in a 2.15-fold, and 1.18-fold, respectively (*p* < 0.05). There was a significant increase in SOD activity in the midgut in the abamectin applied group compared to the control (1.8-fold), while a decrease was observed in the mixture group (2.5-fold) (*p* < 0.05). As for fat body, decreases in SOD activity were detected in the abamectin and mixture applied groups compared to the control (1.66-fold and 1.53-fold, respectively) (*p* < 0.05). As for GPx activity, there was a significant increase in GPx activity in the midgut in the mixture applied group compared to the control (4.68-fold). As for fat body, significant decreases in GPx activity were detected in the Abamectin and mixture applied groups compared to the control (1.40-fold and 1.39-fold, respectively) (*p* < 0.05).

### AChE Enzyme Activity

**Fig. 5:**
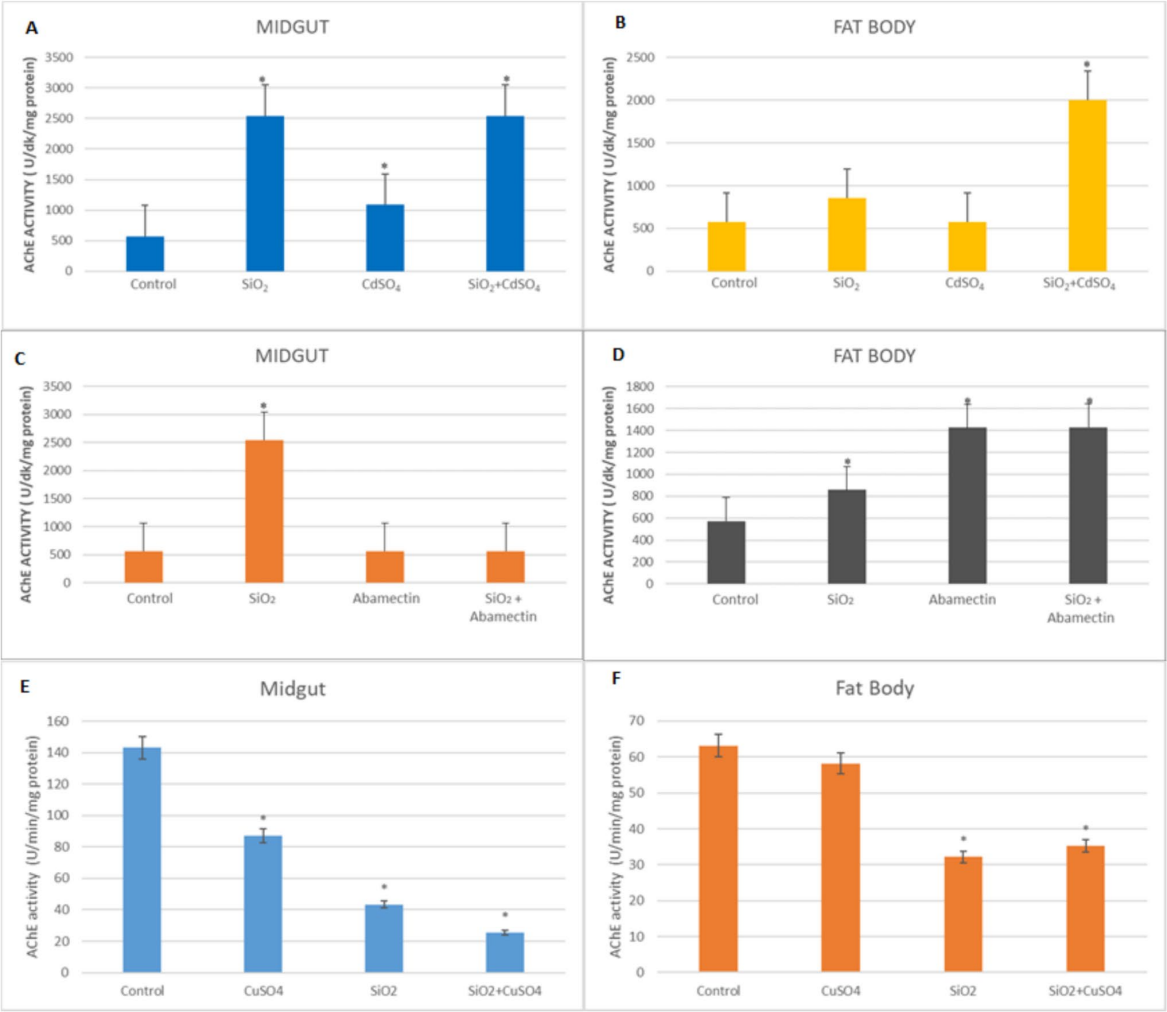
AChE enzyme activities in the midgut and fat body of *G. mellonella* larvae were exposed to SiO_2_ NPs, CdSO_4_, CuSO_4_, and abamectin singly and in mixtures with SiO_2_ NPs (*SNK indicates that there is a statistically notable variation (*p* < 0.05))

The effects of SiO_2_ NPs, CdSO_4_, abamectin singly, and in mixtures on AChE enzyme activity in the midgut and fat body of *G. mellonella* are presented in Fig. 5. Increases were observed in the midgut of SiO_2_ NPs, CdSO_4_, and mixture applied groups according to the control (4.51-fold, 1.92-fold and 4.51-fold, respectively) (*p* < 0.05) (Fig. 5A). As for the fat body, a significant increase was only detected in the mixture applied group, and this increase resulted in 3.50-fold (*p* < 0.05) (Fig. 5B). In the midgut of the abamectin applied groups, there are no significant differences (Fig. 5C). Otherwise, significant increases were observed when larvae were exposed to SiO_2_ NPs and abamectin singly and in mixture in the fat body of the larvae (Fig. 5D). In the midgut of SiO_2_ NPs and CuSO_4_ singly and in mixture applied groups, AChE activities were significantly decreased according to the control (1.65-fold, 3.31-fold, and 5.63-fold, respectively) (Fig. 5E). Also, in the fat body of the SiO_2_ NPs and CuSO_4_ mixture applied group, it was determined that AChE activity was significantly decreased according to the control (1.79-fold) (Fig. 5F).

### GST Enzyme Activity

**Fig. 6:**
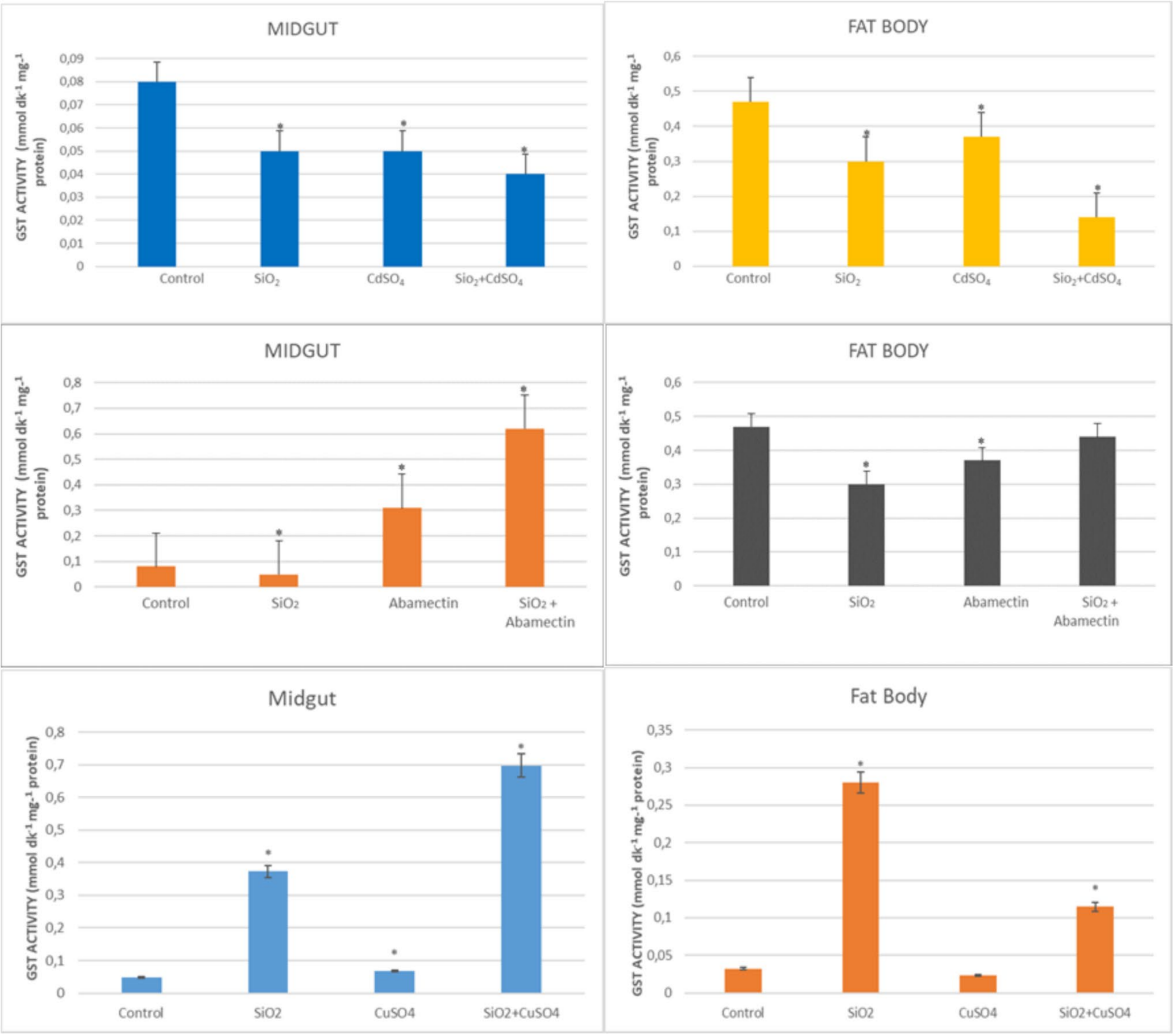
GST enzyme activities in the midgut and fat body of *G. mellonella* larvae were exposed to SiO_2_ NPs, CdSO_4_, CuSO_4_, and abamectin singly and in mixtures with SiO_2_ NPs (*SNK indicates that there is a statistically notable variation (*p* < 0.05))

The effects of SiO_2_ NPs, CuSO_4_, CdSO_4_, abamectin singly, and in mixtures on the GST enzyme activity in the midgut and fat body of *G. mellonella* are presented in Fig. 6. Decreases in GST activity in the midgut were detected in all applied groups compared to the control (1.6-fold, 1.6-fold, and twofold, respectively; *p* < 0.05). Also, decreases in fat body were observed in all applied groups compared to the control (1.57-fold, 1.27-fold, and 3.36-fold, respectively; *p* < 0.05). Increases in GST activity in the midgut were detected in the abamectin singly and in mixture with SiO_2_ NPs applied groups compared to the control (3.88-fold and 7.75-fold, respectively; *p* < 0.05). A decrease in GST activity in fat body was observed in abamectin applied groups compared to the control, and this decrease resulted in 1.27-fold (p < 0.05). In the midgut of the SiO_2_ NPs and CuSO_4_ singly and in mixture applied groups, it was determined that GST activities were increased in all applied groups according to the control (7.65-fold, 1.39-fold, and 14.3-fold, respectively); otherwise, in fat body, the enzyme activity was significantly increased only in SiO_2_ NPs and mixture applied groups (8.88-fold and 3.54-fold) (*p* < 0.05).

### Immunocytotoxic Effects of SiO2 NPs, CuSO4, CdSO4, Abamectin Singly, and in Mixtures

#### Total Hemocyte Count

**Fig. 7:**
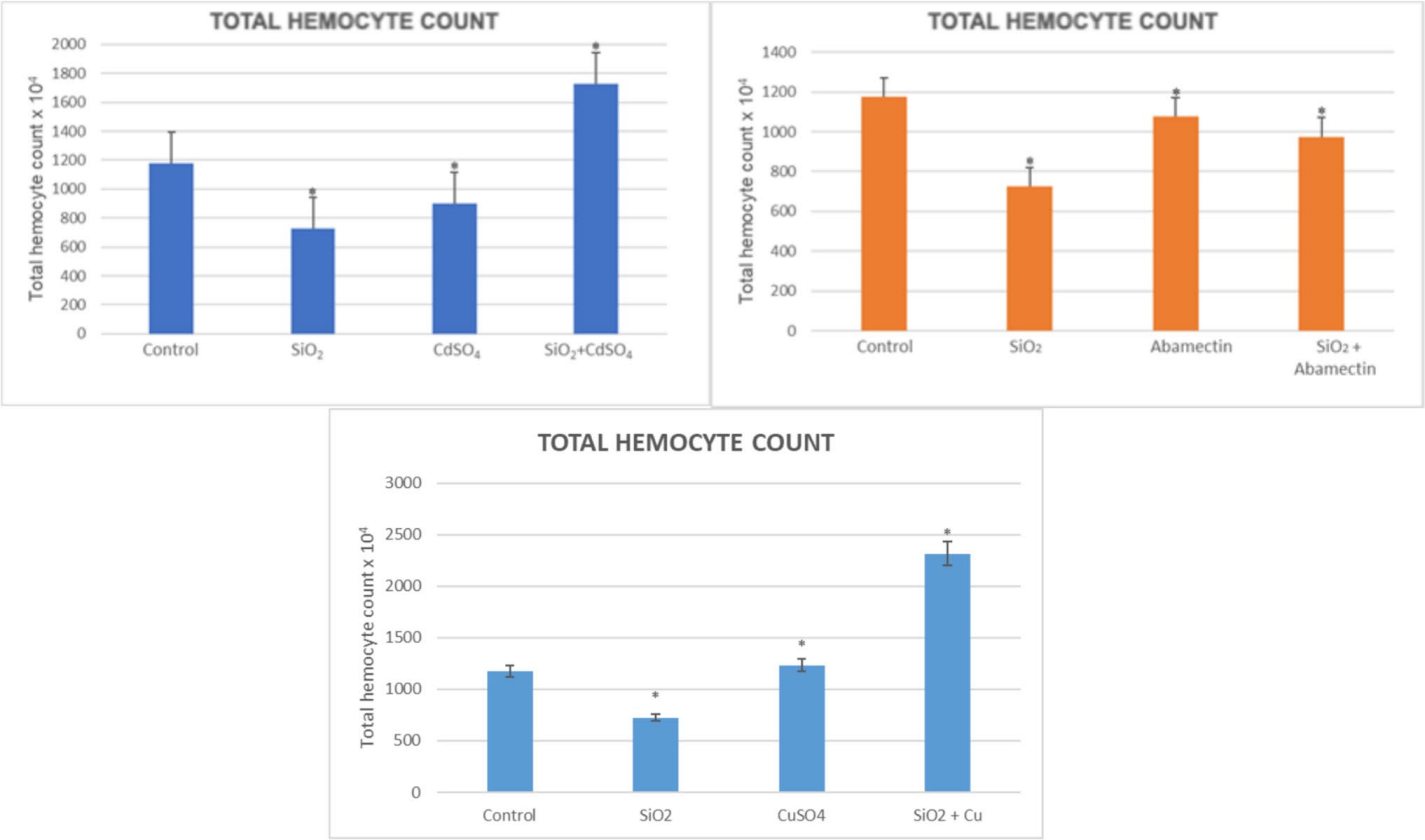
Total hemocyte counts in the hemolymph of *G. mellonella* larvae were exposed to SiO_2_ NPs, CdSO_4_, CuSO_4_, and abamectin singly and in mixtures (*SNK indicates that there is a statistically notable variation (*p* < 0.05))

To evaluate the cytotoxic effects of SiO_2_ NPs and its mixture with environmental pollutants (CdSO_4_, CuSO_4_, and abamectin), we determined total and differential hemocyte count of *G. mellonella* hemocytes. SiO_2_ NPs and CdSO_4_ singly and in mixtures on total hemocyte counts of *G. mellonella* last instar larvae are shown in Fig. 7. Accordingly, it was noted that there was a significant decrease in SiO_2_ and CdSO_4_ singly applied groups compared to the control. However, an increase was detected in the mixture applied group compared to the control (*p* < 0.05). As for the effects of SiO_2_ NPs and abamectin singly and in mixtures on total hemocyte counts, significant decreases occurred in all applied groups compared to the control (*p* < 0.05). Further, THC was significantly increased when larvae exposed to CdSO_4_ and in mixture with SiO_2_ NPs (Fig. 7) (*p* < 0.05).

#### Differential Hemocyte Count

**Fig. 8:**
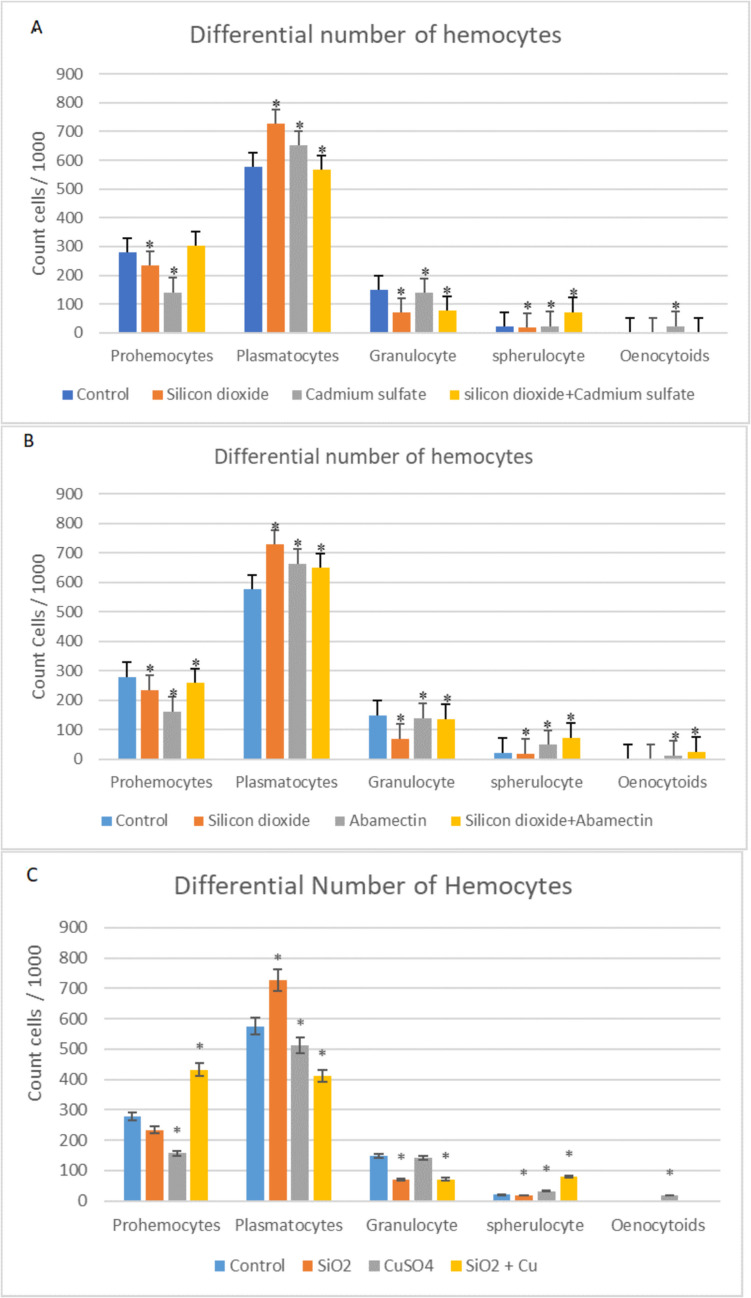
Differential hemocyte counts in the hemolymph of *G. mellonella* larvae exposed to **A** SiO_2_ NPs and CdSO_4_ singly and in mixture **B** SiO_2_ NPs and abamectin singly and in mixture **C** SiO_2_ NPs and CuSO_4_ singly and in mixture (*SNK indicates that there is a statistically notable variation (*p* < 0.05))

The effects of SiO_2_ NPs and CdSO_4_ singly and their mixtures on the differential hemocyte count in the hemolymph of *G. mellonella* last instar larvae are shown in Fig. 8A. In plasmatocyte count, increases were observed in SiO_2_ NPs and CdSO_4_ singly applied groups, while a significant decrease was detected in mixture applied group (*p* < 0.05). As for prohemocyte count, decreases were observed in SiO_2_ NPs and CdSO_4_ singly applied groups, while a significant increase was detected in mixture applied group (*p* < 0.05). A decrease in granulocyte counts was observed in all applied groups compared to the control (*p* < 0.05). When the spherulocyte counts were compared to the control, there was an increase in the application groups of CdSO_4_ and the mixture, but a decrease was observed in the SiO_2_ group. When the oenocytoid counts were examined, an increase was detected in the CdSO_4_ applied group compared to the control (*p* < 0.05). The effects of SiO_2_ NPs and abamectin singly and their mixtures on the differential hemocyte counts in the hemolymph of *G. mellonella* last instar larvae are shown in Fig. 8B. In plasmatocyte count, it was detected that increases were observed in all applied groups compared to the control, whereas in prohemocyte count decreases were detected in all applied groups (*p* < 0.05). Also, decreases were observed in granulocyte count in all applied groups (*p* < 0.05). On the other hand, in spherulocyte count there are increases in abamectin and mixture groups, while spherulocyte count decreased in SiO_2_ NP applied groups (*p* < 0.05). In oenocytoid count, increases were only detected in abamectin and mixture groups (*p* < 0.05). In addition, it was noted that prohemocyte number was increased when larvae were exposed to SiO_2_ NPs in mixture with CuSO_4_; however, it was determined that plasmatocytes and granulocytes were decreased. Further, it was found that spherulocyte number was increased when SiO_2_ NPs were applied in mixture with CuSO_4_. As for eunocytoid number, an increase occurred only in the CuSO_4_ applied group (Fig. 8C) (*p* < 0.05).

#### Phenoloxidase Enzyme Activity in G. mellonella Larvae

**Fig. 9:**
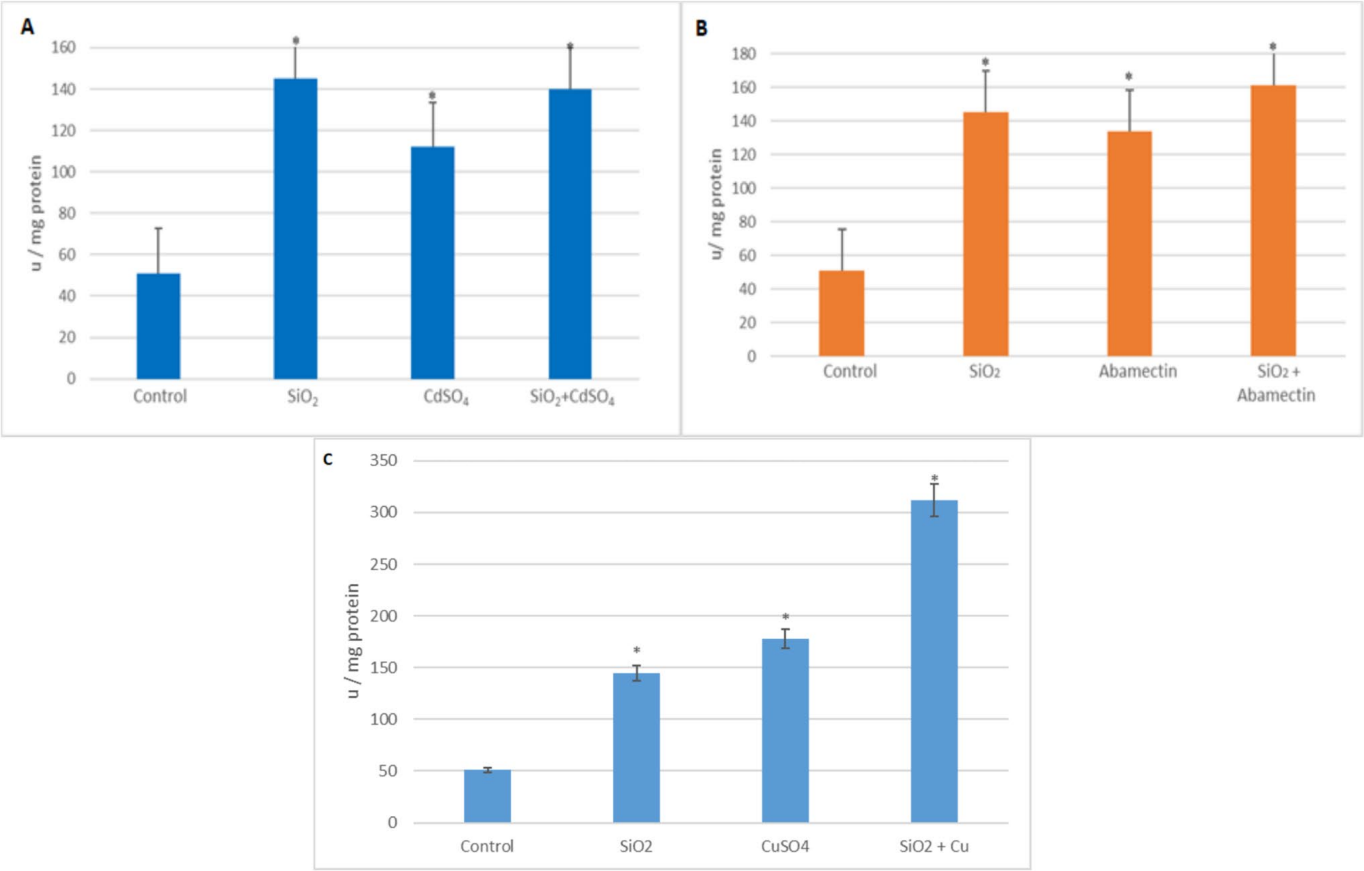
Phenoloxidase activity in the hemolymph of *G. mellonella* larvae was exposed to SiO_2_ NPs, CdSO_4_, CuSO4, and abamectin singly and in mixtures (*SNK indicates that there is a statistically notable variation (*p* < 0.05))

The effects of SiO_2_ NPs and CdSO_4_ singly and in mixture on the phenoloxidase enzyme activity in the hemolymph of *G. mellonella* last instar larvae are presented in Fig. 9-A. In the study, increases occurred in all applied groups compared to the control, and these increases resulted in 2.82-fold, 2.18-fold, and 2.73-fold, respectively (*p* < 0.05). As for the abamectin applied group, the increases were found in 2.82-fold, 2.60-fold, and 3.13- fold, respectively (Fig. 9B). In addition, when larvae were exposed to CuSO_4_ singly and in mixture with SiO_2_ NPs, phenoloxidase activities were also increased according to the control group (2.82-fold, 3.45-fold, and 6.05-fold, respectively) (Fig. 9C) (*p* < 0.05).

#### Apoptotic Index Amount in Larvae

**Fig. 10:**
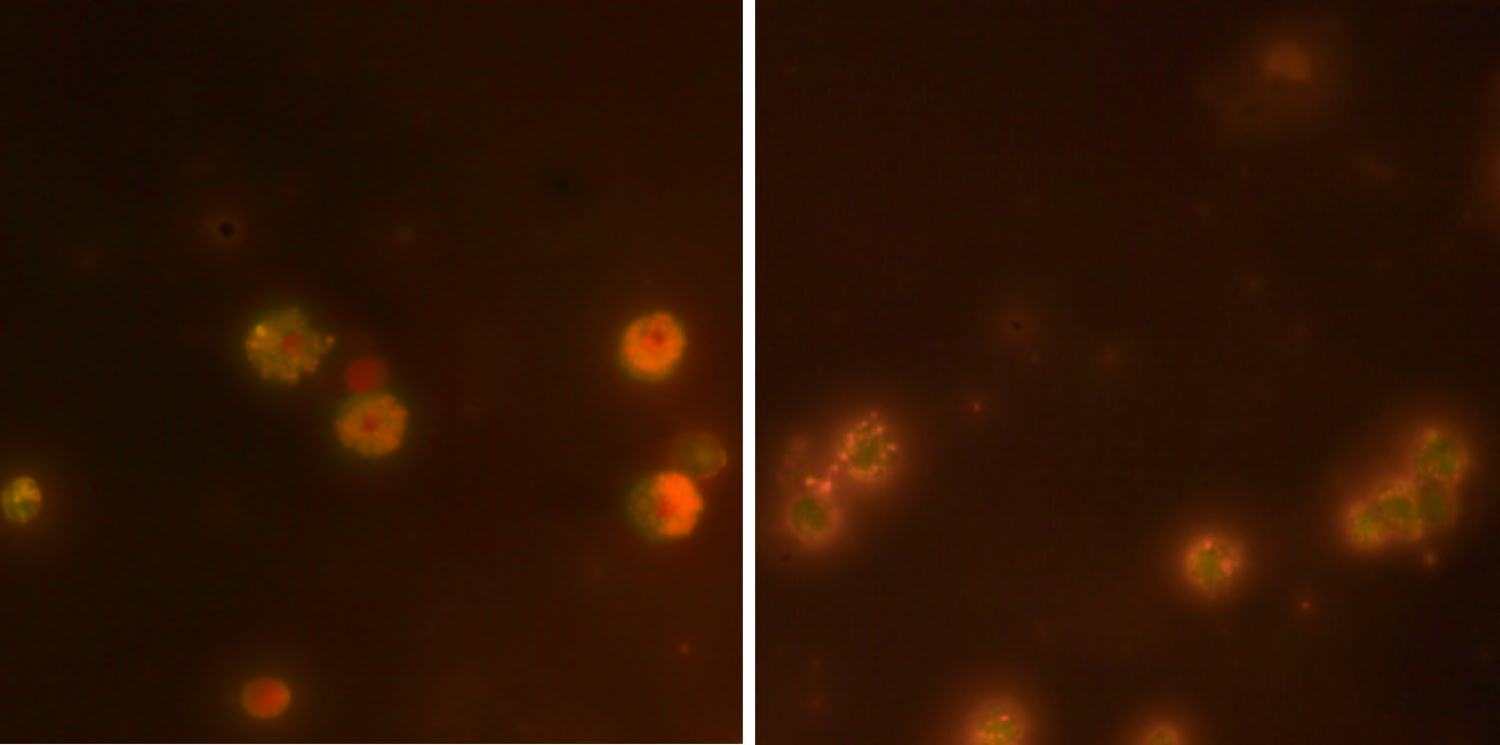
Apoptosis in the hemolymph of *G. mellonella* larvae

In the hemolymph of *G. mellonella* larvae, the apoptotic index was determined according to the cells under the microscope described below (Fig. 10);

These cells are as follows:

1. Live cells: The nucleus is green, while the cytoplasm might be orange or red.
2. Early apoptosis: The cell membrane remains intact, but chromatin condenses and fragments.
3. Late apoptosis is sometimes known as secondary necrosis or apoptotic necrosis. Ethidium bromide penetrates cells with compromised membrane integrity and turns the nucleus orange.
4. Necrosis: The nucleus is orange.

**Fig. 11:**
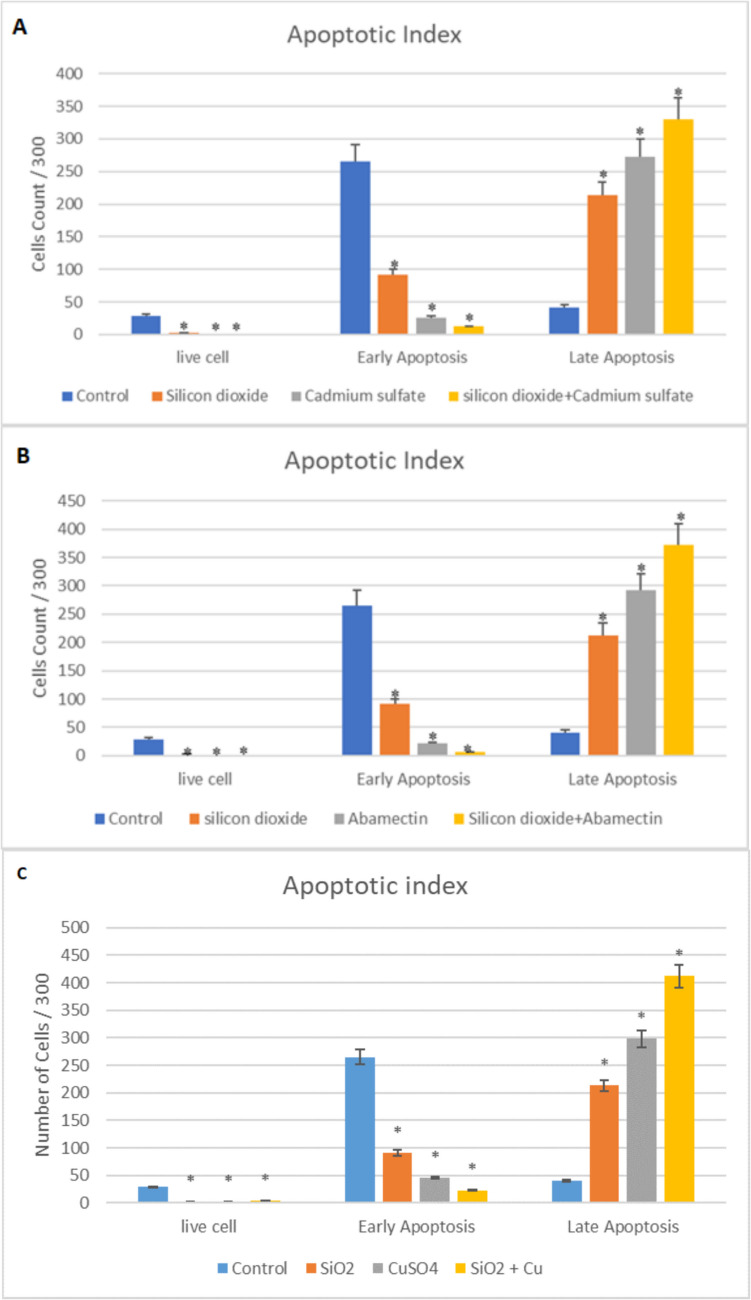
Apoptotic Index in the hemolymph of *G. mellonella* larvae exposed to SiO_2_ NPs, CdSO_4_ (**A**), abamectin (**B**), and CuSO_4_ (**C**) singly and in mixtures (*SNK indicates that there is a statistically notable variation (*p* < 0.05))

The effects of SiO_2_ NPs and CdSO_4_ singly and in mixtures on the apoptotic index of *G. mellonella* are presented in Fig. 11. There were decreases in the count of live cells and early apoptosis in all applied groups (SiO_2_, CdSO_4_, and SiO_2_ + CdSO_4_) compared to the control (*p* < 0.05). Otherwise, significant increases in the late apoptosis were observed in all application groups compared to the control (Fig. 11A) (*p* < 0.05). As for abamectin groups, there were decreases in the count of live cells and early apoptosis in SiO_2_ NPs and abamectin singly and in mixture groups compared to the control (*p* < 0.05). On the other hand, significant increases in the late apoptosis were observed in SiO_2_ NPs and abamectin singly and in mixture groups compared to the control (Fig. 11B) (*p* < 0.05). In addition, significant decreases were observed in live cell and early apoptosis numbers in all applied groups according to the control; however, it was determined that late apoptosis numbers were significantly increased in all applied groups (*p* < 0.05) (Fig. 11C).

## Discussion

In recent years, the unconscious use of nanoparticles and heavy metals in various environments, including industry and agriculture, has led to problems such as disruption of ecological balances and adverse effects on non-target organisms. Furthermore, these nanoparticles, heavy metals, pesticides, and their interactions have the potential to produce significant issues in organismal systems. In this study, the impacts of SiO_2_ NPs, CdSO_4_, CuSO_4_ and abamectin singly and in mixtures on the CAT, SOD and GPx, GST, and AChE enzyme activities in the midgut, and fat body as well as phenoloxidase activity, THC, DHC, and apoptotic index in the hemolymph were investigated.

In insects, xenobiotics such as nanoparticles, chemicals such as heavy metals, pesticides, some microorganisms and radiation that are taken into the body outside of normal metabolic activities cause an increase in free radicals and lead to oxidative stress. This stress causes damage to cellular components and interferes with cellular processes. Free radicals damage macromolecules in cells and activate their defensive mechanisms.

SOD, CAT, and GPx are antioxidants that act as the first defense in neutralizing any molecule that has the potential to become a free radical or that might cause the generation of more radicals [35]. In the study, it was detected that CAT activity increased in both mixture groups in the midgut of the larvae. In the fat body, CAT activity decreased apart from the SiO_2_ NP-applied group. When CuSO_4_ was applied singly and in mixture with SiO_2_ NPs in the midgut of the larvae, it was determined that CAT activities were increased in both singly CuSO_4_ and in mixture with SiO_2_ NP groups. On the other hand, in the fat body, CAT activity was increased only in the mixture applied group. As for the SOD activity in the midgut, it was found that there was a decrease in the groups where SiO_2_ NPs were applied singly and in a mixture with abamectin, but an increase was observed in the groups where CdSO_4_ and abamectin were applied singly and in the SiO_2_ NPs + CdSO4 mixture group. In the fat body, it was also determined that SiO_2_ NPs application decreased SOD activity and increased in the groups in which CdSO_4_ was applied singly and in mixture with SiO_2_ NPs. When abamectin was applied singly and in mixture with SiO_2_ NPs, it was determined that a decrease occurred in SOD activity in the fat body. When CuSO_4_ was applied singly and in mixture with SiO_2_ NPs in the midgut and fat body of the larvae, it was determined that only in the mixture groups significant increases were observed. In GPx activity, it was determined that there was an increase in both groups in the midgut and a decrease in the fat body. When CuSO_4_ was applied singly and in mixture with SiO_2_ NPs in the midgut and fat body of the larvae, significant decreases in GPx activity were determined in both tissues of the mixture applied groups. It is thought that the increased CAT activity may not have been sufficient to eliminate the H_2_O_2_ formed as a result of the oxidative stress caused by the mixture application in the larvae, leading to an increase in GPx enzyme activity. In previous studies, Tuncsoy et al. [28] found that TiO_2_ and CuO NPs enhanced the total protein quantity and antioxidant enzyme activities in *G. mellonella*, indicating an increase in oxidative stress. In another similar study, Emre [25] detected that when *G. mellonella* was exposed to an environmental pollutant, it developed oxidative stress. These induced cellular stress and damage by raising intracellular reactive oxygen species (ROS) levels. They showed that heavy metals like CdSO_4_ impair cellular redox equilibrium, resulting in increased oxidative stress.

The GST enzymes are involved in the second step and are responsible for the modification and conjugation of polar compounds. GST plays a role in protecting cellular integrity, preventing oxidative stress reactions and DNA damage by catalyzing endogenous and exogenous xenobiotics [36]. In the present study, AChE enzyme activities were increased when exposed to SiO_2_ NPs and CdSO_4_ singly and in mixture in the midgut and fat body of the larvae. Also, in the fat body, AChE enzyme activities increased in the SiO_2_ NPs and Abamectin singly and in mixture applied groups. AChE activity was significantly reduced in all application groups of midgut and fat body when CuSO4 was applied alone and mixed with SiO2 NPs. As for GST activities, in all experimental groups, GST activities were decreased apart from SiO_2_ NPs and abamectin singly and in mixture applied groups. On the other hand, GST activity was found to be increased in all application groups of midgut and fat body exposed to CuSO_4_ singly and in mixtures. As a result, it was observed that SiO_2_ NPs increased the toxic effects of both abamectin, CdSO_4_, and CuSO_4_, then led to alterations in detoxification enzymes, AChE and GST in the organism.

Nanoparticles and heavy metals can accumulate in insects’ tissues, resulting in oxidative stress and cell membrane damage. It is known that environmental pollutants such as nanoparticles, pesticides, and heavy metal can be found together in the environment and might be more hazardous if they were singly. Mese et al. [37] investigated the effects of Cu and Zn combinations on *G. mellonella*. Significant decreases in CAT activity were found in the Zn and mixture treated groups. The decreases were linked to the synergistic effects of metal combinations and elevated oxidative stress. Silica nanoparticles and other metal oxide nanoparticles can be incorporated into pesticide formulations, increasing their effectiveness in combating insects. These nanoparticles can kill insects by directly inflicting physical damage or by causing oxidative damage. Moreover, it was also detected that silica nanoparticles can induce synergistic effects when applied together with heavy metals. Guo et al. [38] determined that Cd accumulation in mouse liver increased as a result of a Cd mixture with low concentrations of SiO_2_ NPs and increased the hepatotoxic effect of cadmium. Moreover, Lu et al. [39] applied SiO_2_ NPs and lead in a mixture and as a result, it was determined that while cellular oxidative stress and DNA damage did not occur in lung adenocarcinoma (A549) cells at the non-toxic concentration of silica nanoparticles singly, when applied in a mixture with lead, oxidative stress and DNA damage in cells increased compared to the application of lead singly. As a result, they reported that the mixture application produced a synergistic effect. In another study regarding the synergistic effects of SiO_2_ NPs, Yang et al. [40] reported that the effects of SiO_2_ NPs and methyl mercury singly and in a mixture on human cardiac muscle cells (AC16) caused high toxic effects on cell viability and cell membrane damage. In addition, while ROS caused changes in MDA formation, it caused a decrease in SOD and GSH-Px activities. Moreover, they detected that it caused an increase in cellular apoptosis in heart muscle cells.

Invertebrates have been used as an important model organism in toxicity studies, especially in recent years, due to their ability to be intermediate consumers in food chains. Mostly, the effects of environmental pollutants are measured by their effect on oxidative damage or mortality, but hemocytes are a more convenient tool. Hemocytes have a very important role in the immune system of invertebrates, and insect hemocytes have similar properties to blood cells in vertebrates, making them an important material for immunological studies [41]. In this study, THC decreased when exposed to SiO_2_ NPs, CdSO_4_, and abamectin singly. Nonetheless, it was determined that THC increased in the groups where CdSO_4_ and abamectin were applied in mixture with SiO_2_ NPs. By causing oxidative stress in the cells, both SiO_2_ NPs, CdSO_4_ and abamectin, may have caused damage or death to the hemocytes. It was known that oxidative stress can cause damage to cell membranes, DNA, and proteins and can lead to cell death. On the other hand, in the SiO_2_ + CdSO_4_ mixture group, it may have caused an increase in the count of hemocytes by increasing the activity of the immune system. This can be interpreted as the body’s defense mechanism against toxic substances. Besides, when CuSO_4_ applied both singly and in mixture with SiO_2_ NPs, THC was significantly increased. Similar results were determined in our previous study when different concentrations of CuO NPs to the diet of *G. mellonella*, THC was significantly increased [37]. As for DHC, it was determined that the count of plasmatocytes increased in the groups in which SiO_2_, CdSO_4_, and abamectin were applied singly and in the groups in which abamectin was applied in mixture with SiO_2_ NPs. On the other hand, it was analyzed that plasmatocytes significantly diminished in the groups exposed to CuSO_4_ singly and in mixture with SiO_2_ NPs. Plasmatocytes are blood cells capable of phagocyte [42]. It is thought that the increase in the count of plasmatocytes in these treated groups occurred in order to destroy foreign substances in the blood circulation. Moreover, it is thought that the reason for this increase is that plasmatocytes accumulate metals on the hemocoel wall with their ability to adhere, and this may be related to the blood cell’s resistant effect against metals [43]. Besides, the decrease in plasmatocytes when CuSO_4_ was applied might be due to cellular lysis [44]. It was also determined that the count of prohemocytes decreased in SiO_2_ NPs and CdSO_4_ applications singly, while the count of prohemocytes increased when applied as a mixture. This increase suggests that SiO_2_ NPs increase the toxic effect of Cd, and the count of prohemocytes, which are hematopoietic cells, increases for defense purposes. On the other hand, a decrease was observed in the groups in which abamectin was applied singly and in mixture with SiO_2_ NPs. In previous studies, it was reported that insecticides decreased the count of prohemocytes [45]. It is thought that prohemocytes may have differentiated into plasmatocytes in order to phagocytize foreign substances entering the body, and as a result, the percentage of prohemocytes in the blood circulation may have decreased [28, 46]. In the study, decreasing prohemocyte and increasing plasmatocyte counts in the treatment groups support this. Granulocytes decreased in all applied groups. It is thought that this blood cell decreased because granulocytes are responsible for phagocytosis and can digest foreign substances taken in by hydrolytic enzymes. Besides, spherulocytes increased in all treatment groups. Although many comments have been made about the regulation of melanization, phagocytosis, coagulation, and cell adhesion of spherulocytes, it has not been clarified yet [47]. As for eunocytoids, it was determined that an increase occurred in the groups where CdSO_4_ and abamectin were applied as a mixture with SiO_2_ NPs. Eunocytoids contain the enzyme phenoloxidase, which is responsible for melanization in the immune system. Studies have reported that phenoloxidase is synthesized in oenocytoids and released into plasma when they are lysed [48]. It is suggested that the increase in oenocytoids in the groups treated with CdSO_4_ and abamectin singly or in combination is due to the toxic effects of these substances. This is supported by the fact that the same application groups in the study showed an increase in phenoloxidase enzyme activity. In a study conducted by Çoğal et al. [49], it was determined that there was a visible decrease in the total hemocyte counts of *G. mellonella* last instar larvae applied with Al_2_O_3_ NPs compared to the control group. Considering the important role of hemocytes in insect immunity, these results indicate that Al_2_O_3_ NPs have suppressive effects on the immune system of *G. mellonella*. In addition, it was determined that there was a decrease in the count of granulocytes, prohemocytes, spherulocytes, and oenocytoids, which are hemocyte types, while there was an increase in the count of plasmatocytes. It is thought that the reason for the increase in plasmatocytes is that these cells accumulate metals by adhering them to the hemocoel wall and tend to resist metals [43]. Eskin [50] conducted a study with SiO_2_ NPs and reported that it has a considerable influence on the insect’s total hemocyte count and vitality. SiO₂ NPs were found to be 50% deadly at a dosage of 411.93 μg/10 µL. Greater doses (100 and 180 μg/10 µL) resulted in considerably greater cell death rates than the control group. Adult growth time and longevity were considerably decreased in larvae fed low concentrations of SiO_2_ NPs.

Phenoloxidase (PO) is a typical metal enzyme, which requires metal ions as prosthetic groups to enable the full exertion of its activity and is essential for insect immunological system [51]. It is known that physical injury or tissue damage can also stimulate phenoloxidase activity. In the event of an injury, the insect’s immune system responds swiftly and activates defensive systems surrounding the affected region. This is linked to the insect’s increased immunological response to speed up wound healing or cope with stress. Temperature fluctuations, environmental pollutants, and other stressors can all enhance phenoloxidase activity. Xu et al. [52] showed that ZnO NPs can trigger a protective immune response in *G. mellonella*. This response involves an increase in phenoloxidase activity. In an another research conducted by Wu and Yi [53], it was analyzed that environmental pollutants such as chromium (Cr) and lead (Pb) have high toxicity on immune and antioxidant system of *G. mellonella*. Antioxidant enzymes (CAT, SOD, and peroxidase), THC, and phenoloxidase activities were increased with increasing concentrations of dietary Cr and Pb. Based on our results, it can be concluded that in all mixture applied groups due to high levels of oenocytoids, phenoloxidase activities were increased. Thus, hemocytes may have disintegrated in the presence of SiO_2_ NPs, abamectin and CuSO_4_, CdSO_4_, and their mixtures, leading to the release of phenoloxidase enzyme into hemolymph. As for apoptotic index, there was a decrease in the count of living cells and early apoptosis in SiO_2_, CuSO_4_, and CdSO_4_ singly and in mixture applied groups compared to the control, and an increase in the count of late apoptosis was observed in the same groups. There was also a decrease in the count of live cells and early apoptosis in SiO_2_ NPs and abamectin singly and in mixture applied groups compared to the control, and an increase in the count of late apoptosis was observed in all application groups compared to the control. The reason for the gradual decrease in living cells is their transformation into early and late apoptosis due to the toxic effects of nanoparticles, heavy metals, and pesticides. The reason for the gradual decrease in early apoptosis can be considered as its transformation into late apoptosis. In a research conducted by Eskin et al. [54] with CuO NPs, it was determined that there was an increase in apoptotic counts in *G. mellonella* and no necrotic death occurred.

According to studies done with several insect species including *G. mellonella* larvae, blood cells in insects exposed to environmental contaminants such as nanoparticles, heavy metals, and pesticides change the morphological, histochemical, biochemical, and immunological defense systems. Knowing the observed toxic effect mechanism of silicon dioxide nanoparticle (SiO_2_ NP), CdSO_4_, abamectin, and their mixtures on insects is thought to allow the development of new chemical methods in the fight against harmful insects that have less adverse impact on non-target organisms and the environment. Due to the advancement of nanotechnologies, NPs are increasingly being employed and dispersed into the environment, either unintentionally or purposely. Consequently, humans may be exposed to increasingly large counts of these particles. Furthermore, NPs can enter the body through oral exposure as well as inhalation, and co-ingestion of NPs with other contaminants such as pesticides can also have a negative effect on human health, particularly on the gastrointestinal tract [55–57]. Although they are constantly exposed to many pollution sources, the interactions between contaminants and the consequent cumulative toxicity have not received adequate attention in the literature. However, NPs discharged into the environment may interact with other contaminants or absorb them on their surfaces, allowing them to enter the body [58]. Even if the toxicity of individual compounds is well understood, it has been proposed that when such substances are combined, unanticipated detrimental impact may arise [59, 60], implying a collective toxicity. A co-exposed contaminant may impact the cell membrane (in terms of fluidity, hydrophobicity, physical integrity or permeability,), increasing NP internalization and toxicity [4, 5]. According to Deng et al. [61], NPs can be co-exposed to a wide range of compounds, including organic contaminants, metal/metalloid ions, inorganic ligands, and other NPs. The ability of NPs to adsorb a co-pollutant can have a significant impact on the toxicity of the NP or pollutant, particularly by promoting pollutant entrance into the body via the absorption of NPs-pollutant complexes. Once inside, complex contaminants can be released into the body, increasing their concentration and consequently bioavailability and toxicity. According to Lu et al. [39], harmful substances can be adsorbed on the surface of nanoparticles, allowing them to benefit from the carrier effect and reach the alveoli, causing more damage. Nonetheless, other pollutants can lessen NP toxicity by scavenging the ROS they create. Contaminants, on the other hand, have the potential to exacerbate the negative effects of nanoparticles by producing more ROS [5].

In conclusion, we determined that significant decreases and increases in the activities of Cyt P450, an important detoxifying enzyme, and GST and AChE, enzymes responsible for neurotransmission, were detected in response to changes in the amount of silica nanoparticles, CdSO4, and abamectin, as well as CAT, SOD, and GPx, indicators of oxidative stress. The use of sublethal doses in research is considered an ecologically acceptable method of pest control. The determination of LD_50_ values helps to prevent the overuse of nanoparticles, leading to better results. Using nanoparticles with low LD_50_ values in combination has several advantages, including minimizing air pollution, slowing down the development of resistance, and reducing costs. Although there were variations between tissues in the research, changes in antioxidant enzymes and detoxification enzymes were generally found as a result of the mixture. As a result, we concluded that SiO_2_ NPs may absorb CdSO_4_ and abamectin and increased the toxicity of these environmental pollutants.

## Data Availability

No datasets were generated or analysed during the current study.
